# Impacts of nutrient loading and fish grazing on the phytoplankton community and cyanotoxin production in a shallow tropical lake: Results from mesocosm experiments

**DOI:** 10.1002/mbo3.1278

**Published:** 2022-04-27

**Authors:** Mathias K. Ahoutou, Eric Kouamé Yao, Rosine Y. Djeha, Mamadou Kone, Kevin Tambosco, Charlotte Duval, Sahima Hamlaoui, Cécile Bernard, Marc Bouvy, Benjamin Marie, Bernard Montuelle, Marc Troussellier, Felix K. Konan, Julien Kalpy Coulibaly, Mireille Dosso, Jean‐François Humbert, Catherine Quiblier

**Affiliations:** ^1^ Institut Pasteur d'Abidjan Abidjan Côte d'Ivoire; ^2^ Université Jean Lorougnon Guédé UFR Environnement Daloa Côte d'Ivoire; ^3^ iEES Paris, INRAE‐Sorbonne Université Paris France; ^4^ MNHN, UMR 7245 Molécules de Communication et Adaptation des Micro‐organismes Paris France; ^5^ UMR MARBEC, IRD‐Université de Montpellier Montpellier France; ^6^ UMR CARRTEL, INRAE‐Université de Savoie Thonon‐Les‐Bains France; ^7^ Université Paris Cité UFR Sciences du vivant Paris France

**Keywords:** biomanipulation, eutrophication, freshwater lagoon, Ivory Coast, mesocosm experiment, phytoplankton

## Abstract

Given the increasing eutrophication of water bodies in Africa due to increasing anthropogenic pressures, data are needed to better understand the responses of phytoplankton communities to these changes in tropical lakes. These ecosystems are used by local human populations for multiple purposes, including fish and drinking water production, potentially exposing these populations to health threats if, for example, an increase in toxic cyanobacterial blooms is associated with increasing eutrophication. To test the short‐term response of the phytoplankton community to the addition of nutrients (phosphorus and nitrogen, alone or in combination) and Nile tilapia, we developed an in situ mesocosm experiment in a freshwater lagoon located near Abidjan (Ivory Coast). We found that phytoplankton growth (estimated by chlorophyll‐a quantification) was highly stimulated when both nitrogen and phosphorus were added, while there was no clear evidence for such colimitation by these two nutrients when considering their concentrations in the lagoon. Phytoplankton growth was accompanied by significant changes in the diversity and composition of this community and did not lead to an increase in the proportions of cyanobacteria. However, the addition of fish to some mesocosms resulted in a drastic decrease in phytoplankton biomass and a dominance of chlorophytes in this community. Finally, these experiments showed that the addition of nitrogen, alone or combined with phosphorus, stimulated microcystin production by cyanobacteria. In addition, no evidence of microcystin accumulation in the fish was found. Taken together, these data allow us to discuss strategies for controlling cyanobacterial blooms in this tropical ecosystem.

## Graphical abstract

Low‐cost aquatic mesocosms easy to transport and construct have been developed to study the short responses (7 days) of phytoplankton to an increase in nutrient concentrations and the addition of fish in an African freshwater lagoon. A simultaneous phosphorus–nitrogen colimitation of the phytoplankton growth was evidenced and fish addition has led to drastic changes in the biomass and composition of the phytoplankton community. Finally, nitrogen addition has stimulated microcystin production by cyanobacteria.

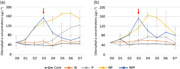

## 1 INTRODUCTION

African surface freshwater ecosystems have experienced increasing anthropogenic pressures due to human population growth on this continent, and the population of sub‐Saharan Africa is projected to double by 2050 (UNECA, 2016). In addition to pollution from pharmaceuticals and pesticides (Fekadu et al., 2019; Olisah et al., 2020), these pressures have led to the nutrient enrichment (eutrophication) of numerous water bodies ranging from small water bodies (e.g., Fetahi, 2019; van Ginkel, 2012) to large lakes, such as Lake Victoria, East Africa (e.g., Olokotum et al., 2020). One of the main consequences of eutrophication in freshwater ecosystems is the development of cyanobacterial blooms, which have numerous impacts on ecosystem functions (Filstrup et al., 2014) and their uses (Carvalho et al., 2013; Sanseverino et al., 2016). Among them, the production of drinking water and/or the multiple uses of water by local human populations could be particularly impacted by the recurrent proliferation of potentially toxic cyanobacteria.

Numerous studies have been performed to understand and solve the eutrophication problem (e.g., Chorus & Spijkerman, 2021; Schindler, 2012; Smith & Schindler, 2009). They have shown that the key nutrients involved in the eutrophication process are nitrogen (N) and phosphorus (P). In general, N is considered the primary limiting nutrient in marine ecosystems and P is the main limiting nutrient in freshwater ecosystems. However at low latitudes, several authors suggest that the N limitation of the phytoplankton growth is more frequent than the P limitation in freshwater ecosystems (Talling & Lemoalle, 1998; Maberly et al., 2020), however, there are also numerous examples suggesting phytoplankton growth is P limited (e.g., Gikuma‐Njuru et al., 2013; Kalff, 1983; Melack et al., 1982).

When nutrients are nonlimiting to phytoplankton growth, other processes, such as light limitation, may control phytoplankton biomass. For example, it has been shown in several African aquatic ecosystems that an increase in mineral particles and/or terrestrially derived dissolved organic matter in the water column during the rainy season limits light penetration with a negative consequence on phytoplankton growth (Fee et al., 1996; Gebrehiwot et al., 2020; Loiselle et al., 2008; Nyakoojo & Byarujali, 2010; Okech et al., 2018; Olson et al., 2020). Furthermore, self‐shading may also limit phytoplankton growth in eutrophic and hypereutrophic freshwater ecosystems where phytoplankton biomasses reach very high levels (Huisman & Weissing, 1994). Consequently, it is challenging to predict how a given freshwater ecosystem will respond to an increase in its nutrient load.

Phytoplankton growth can also be controlled directly (grazing) or indirectly (trophic cascades) by zooplankton and fish communities (see, e.g., the reviews by Ger et al., 2016; Sommer et al., 2012). Most of the data available on this issue are for temperate freshwater ecosystems, but it has been shown that the food webs in shallow lakes display significant differences between subtropical and temperate areas (Iglesias et al., 2017). For example, omnivory is a dominant characteristic of subtropical and tropical fish communities, and omnivorous fish upregulate and downregulate phytoplankton, particularly by the direct ingestion of these microorganisms or by cascade effects (e.g., Ferrao‐Filho et al., 2020; Lazzaro, 1997; Lazzaro et al., 2003; Mayer, 2020; Moustaka‐Gouni & Sommer, 2020). More specifically for cyanobacteria, it has been shown in tropical areas that some fish species can control their blooms (Torres et al., 2016; Yi et al., 2016).

To test the potential responses of phytoplankton communities to an increased nutrient load and/or to food web manipulations, various experimental approaches have been developed, from the use of microcosm and mesocosm experiments to the manipulation of whole lakes (Amorim & Moura, 2020; Carpenter et al., 2001; Filiz et al., 2020; Quiblier et al., 2008; Schindler, 1974, 2012; Xu et al., 2015). These types of experiments have also been widely used in ecotoxicological studies (e.g., Shaw & Kennedy, 1996; Solomon & Hanson, 2012; Vera et al., 2010). Mesocosms represent a good compromise between small volume experiments and lake manipulation because (i) they allow the performance of controlled and replicated experiments in large water volumes under the same temperature and light conditions as those in natural aquatic ecosystems and (ii) mesocosms can be filled with water from the studied freshwater ecosystems and thus contain populations and communities from multiple trophic levels. However, mesocosm experiments are difficult to maintain for long periods, which limits their use for predicting the long‐term responses of whole lakes (Schindler, 2012).

In the framework of a research program dealing with the freshwater lagoon Aghien (Ivory Coast), we aimed to assess the potential impact of increasing pollution by nutrients (phosphorus and nitrogen) and local omnivorous fish on its phytoplankton community. This lagoon, which provides multiple ecosystem services for local populations, is located in the peri‐urban area of Abidjan city, and consequently is being affected by the growing urbanization of its watershed. The first objective of this study was to test whether the addition of nutrients (P and N) leads to changes in phytoplankton biomass and composition. Inside this community, special attention has been given to cyanobacteria and their toxins because of the potential health risks associated with them (Chorus & Welker, 2021). The second objective of this study was to evaluate the impact of the ability of locally farmed fish (*Oreochromis niloticus*) to affect phytoplankton biomass and composition and potentially accumulate cyanotoxins in their tissues. This study was performed by using a simple and low‐cost mesocosm system and involved local populations to raise their awareness of the water pollution issue.

## 2 MATERIALS AND METHODS

### 2.1 Study site

Lagoon Aghien (5°22' N to 5°26' N and 3°49' W and 3°55' W) is a freshwater ecosystem located in the district of Abidjan in the south of the Ivory Coast (see the map in Ahoutou et al., 2021). This lagoon is characterized by an area of 19.5 km^2^, a perimeter of 40.7 km, a volume of 75 km^3,^ and a maximum depth of 10 m (Koffi et al., 2019). In a recent study performed by Ahoutou et al. (2021), the authors showed that the lagoon is hypereutrophic, as evidenced by the high concentrations of total phosphorus (TP) (>140 µg L^−1^), total nitrogen (TN) (>1.36 mg L^−1^), and chlorophyll‐a (Chl‐a) (26‐167 µg L^−1^). Moreover, the phytoplankton community in the lagoon is dominated by genera that are typical of eutrophic ecosystems such as *Peridinium* and several cyanobacterial genera, *Raphidiopsis, Microcystis*, and *Dolichospermum*, known to potentially produce cyanotoxins (Ahoutou et al., 2021).

This lagoon is already used for multiple purposes (washing dishes and clothes, bathing, fishing, and obtaining water for consumption) by local populations (Effebi et al., 2017), and the construction of a water treatment plant in Débarcadère started in 2021 to provide drinking water to part of the population of the Abidjan agglomeration from 2023 to 2024.

Mesocosm experiments were performed at two different stations, Débarcadère and Aghien‐Télégraphe (see Ahoutou et al., 2021), to detect potential spatial variations in the responses of the phytoplankton communities to the various treatments tested. Débarcadère is a very shallow station located at the western end of the lagoon, close to the mouths of its two main tributaries (the rivers Bété and Djibi); Aghien‐Télégraphe is located on the northern shore of the central part of the lagoon. During the experiment, there was a marked daily wind blowing at the Aghien‐Télégraphe station but not at the Débarcadère station.

In the two villages located close to these stations, we organized a meeting with the chiefs to present the project and to ask them for their permission before beginning to work. In addition, during the experiments, some people from the villages visited our facilities, and small experiments were organized with the teachers of two schools (one per village) to explain our work to the children. A few months after the end of the experiments, we organized one meeting per village to present the main findings from these experiments.

### 2.2 Experimental set‐up

**Figure 1:**
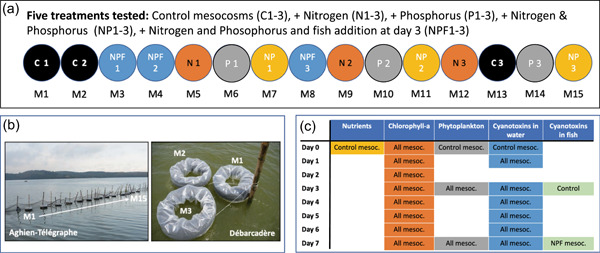
Design of the mesocosm experiment at the two stations (Débarcadère and Aghien‐Télégraphe (a), the installation of the mesocosms at Aghien‐Télégraphe and Débarcadère stations (b) and the water and fish sampling for the analyses during the experiment (c)

The mesocosm experiments were carried out in February 2018 during the long dry season. To limit the cost of these mesocosms so that they could be reproduced by local teams, very inexpensive materials and a simple assembly method were implemented. The mesocosms were constructed with transparent polyethylene bags (200 L capacity) attached to plastic rings (hula hoop) and supported by motorcycle or car tubes, enabling them to float. Fifteen mesocosms numbered from 1 to 15 were installed and filled with 160 L of lake water at each station (Figure 1a). The positions of the 15 mesocosms in the experimental setup (from M1 to M15) were randomly determined to avoid potential biases due to their locations (Figure 1a). In Débarcadère, the mesocosms were tied in groups of three to wood piles located 20 m away from the shore (2 m depth), while in Aghien‐Télégraphe, the mesocosms were attached in increasing order to a wooden barrier located 10 m away from the shore (1.6 m depth) (Figure 1b). The main characteristics of these two stations are provided in Table A1.

The design of the experiment at each site was as follows (Figure 1a):

– Three mesocosms (C1, C2, & C13) were used as controls (C treatment),
– Three mesocosms (N5, N9, & N12) were supplemented only with nitrogen (16 ml of the N solution) (N treatment), and the N‐solution used for nutrient enrichment was formed from NaNO_3_ with an N concentration = 11.53 mg ml^−1^.
– Three mesocosms (P6, P10, & P14) were supplemented only with soluble phosphorus (16 ml of the P solution) (P treatment), and the P‐solution was formed from KH_2_PO_4_ with a P concentration = 1.59 mg ml^−1^.
– Three mesocosms (NP7, NP11, & NP15) were supplemented with nitrogen and phosphorus (16 ml of the N solution and 16 ml of the P solution) (NP treatment),
– Three mesocosms (NPF3, NPF4, & NPF8) were supplemented with nitrogen and phosphorus (16 ml of the N solution and 16 ml of the P solution), and five fish (*Oreochromis niloticus*) were added to each of them on Day 3 (D3) (NPF treatment). All fish used in the experiment were a mix of males and females; they were purchased at a fish farm located in the lagoon. They were hand‐selected with the goal of them having similar lengths and masses of approximately 120 mm and 30 g, respectively. Five additional fish were measured, weighed, and dissected at D3. They were not added to a mesocosm and used as a control. At the end of the experiments at D7, all the alive fish (*n* = 34, one fish died) were measured, weighed, and dissected following ethical animal concerns and regulations. For each individual, the liver, muscles, and intestines were collected, quickly frozen by using liquid nitrogen, and further stored at −80°C until cyanotoxin analysis.

For the nutrient addition, the volume of P solution added to the P, NP, and NPF mesocosms was calculated by multiplying the initial soluble reactive phosphorus (SRP) concentration by 10. The volume of N solution added to the N, NP, and NPF mesocosms was calculated with the goal of maintaining the N/P Redfield ratio between the volume of the solutions added.

For all the analyses that were performed during the experiment (see the following paragraphs), the schedule of the water and fish samplings in the mesocosms is provided in Figure 1c. Water sampling and probe measurements were performed in mesocosms between 7:00 am and 9:00 am at Aghien‐Télégraphe station and between 10:00 am and 2:00 pm at Débarcadère station.

### 2.3 Nutrient analyses

Nutrient analyses were performed at D0 in the lagoon water used to fill the mesocosms and at D3 and D7 in all the mesocosms, following the instructions of the LCK cuvette test systems provided by the manufacturer (Hach® Company). The preparation of samples for the dissolved nutrient analyses was performed using cuvette test systems LCK 304, 341, 339, and 349 for ammonium (NH_4_^+^), nitrites (NO_2_^−^), nitrates (NO_3_^−^), and SRP, respectively, after the water samples were filtered through nylon membranes (Whatman^TM^, porosity 0.45 µm, diameter 47 mm). The preparation of the samples for the TN and TP analyses was performed using unfiltered water samples, with LCK cuvette test systems LCK 138 and 349, respectively. The protocols for the TN and TP measurements included a digestion step at a high temperature, performed with a HACH HT 2500 thermostat. Finally, colorimetric determinations of the concentrations were obtained for all the nutrients using a HACH DR6000 UV‐VIS spectrophotometer.

### 2.4 Multiparameter probe measurements

A YSI EXO2 multiparameter probe (YSI Inc.) was used for in situ measurements in the mesocosms of water temperature, turbidity, oxygen (O_2_) concentration/saturation, and fluorescent dissolved organic matter (fDOM) concentrations.

### 2.5 Phytoplankton biomass and composition

#### 2.5.1 Chl‐a concentrations

On each sampling date, 100 ml of lake water was filtered through glass microfiber filters (Whatman® GF/C) in triplicate. Filters were frozen and stored at −20°C. Chl‐a and phaeopigment extraction were performed in 10 ml 90% aqueous acetone solution at 4°C for 24 h. Then, the solution was centrifuged at 2000 rpm for 15 min. The supernatant was used to calculate pigment concentrations by spectrophotometry at 750 nm (for correction linked to the turbidity of the extract) and 665 nm before and after acidification. Acidification, which allows the destruction of Chl‐a but not that of phaeopigments, was performed by adding two drops of 5% (v/v) hydrochloric acid to the extracts. The spectrophotometric analyses were carried out using a Hach Lange DR 6000 UV‐VIS spectrophotometer.

The Chl‐a concentrations were calculated by using the following formula (Lorenzen, 1967):

$$[\mathrm{Chl}-a](\mathrm{\mu g}\;\cdot \;L^{-1})=26,7\left(E1–E2\right)\times V/(L\times V_{s}),$$

where *E*1 is the absorbance (OD_665_‐–OD_750_) before acidification; *E*2 is the absorbance (OD_665_–OD_750_) after acidification; *V* is the  volume of 90% acetone in ml; *L* is the length of the optical path; *V*_s_ is the volume of filtered sample (expressed in liters).

#### 2.5.2 Identification and counting of the phytoplankton genera

For each water sample, 100 ml was collected and fixed in a formaldehyde solution (5% final concentration). The identification and cell counts of the phytoplankton genera were performed on an inverted microscope (Nikon Eclipse TS100) using the Utermöhl method (Utermöhl, 1958) according to the AFNOR 15204 standard. For each sample, at least 500 counting units (cells, colonies, or filaments/trichomes) were counted under the microscope on a minimum of 30 fields of view randomly selected and distributed over the entire surface of the bottom of the counting chamber. For *Microcystis*, two categories of colonies were considered corresponding to their diameters (< or >200 µm). The mean number of cells per category was estimated under an upright microscope by counting the number of cells in 30 colonies per category after the colonies were gently spread between slides and coverslips. For filamentous cyanobacteria (*Aphanizomenon*, *Raphidiopsis*, *Limnothrix*, *Lyngbya*, *Oscillatoria*, *Planktothrix*, and *Pseudanabaena*), filament lengths of 100 µm were considered, and the mean cell number per 100 µm of filament was estimated on 30 filaments per genus according to the method described by Catherine et al. (2017). Finally, for all genera, the results of the cell counts were expressed as numbers of cells per milliliter. The mean cell biovolumes were estimated for the nine most abundant genera (*Microcystis*, *Raphidiopsis*, *Limnothrix*, *Oscillatoria*, *Dolichospermum*, *Scenedesmus*, *Staurastrum*, *Aulacoseira*, and *Peridinium*) by the measurements of 30 cells per genus. For all the other genera, we used the standard cell biovolume values provided in the HELCOM PEG Biovolume reports (https://helcom.fi/helcom-at-work/projects/peg/).

The richness and diversity (Shannon index) of the phytoplankton communities were estimated at the genus level.

### 2.6 Identification and quantification of cyanotoxins in phytoplankton communities and fish

#### 2.6.1 Cyanotoxin extraction from phytoplankton

For each sample, 200 ml of water was filtered on polycarbonate filters (Whatman® Nuclepore, 0.4 µm porosity) and frozen at −80°C. After thawing, the filters were placed in 15 ml sterile glass vials to which 4 ml of 75% methanol was added. Samples were sonicated on ice with an ultrasound probe (Sonics Vibra Cell) (100% amplitude, 130 W, 20 kHz) for 3 min with 30‐s pulse intervals to facilitate toxin extraction. The extracts were centrifuged at 3000 rpm for 15 min, and the supernatants were collected in 15 ml glass tubes and stored in a refrigerator at 4°C. The resulting pellets underwent a second extraction using 2 ml 75% methanol in an ultrasonic bath containing ice for 15 min and then centrifuged again to ensure that all toxins were extracted. These second supernatants were added to the first and centrifuged again at 12,000 rpm for 5 min. One hundred microliters of these supernatants were frozen at −80°C until cyanotoxin analysis.

#### 2.6.2 Cyanotoxin extraction from fish

The method of Manubolu et al. (2018) was used to extract cyanotoxins from the fish tissues. For each fish sample, 100 mg of fresh liver, muscle, and intestine tissues were used for cyanotoxin extraction. The muscle extracts were previously freeze‐dried and underwent ball milling. All samples were extracted twice with 5 ml BuOH:MeOH:H_2_O (1:4:15). They were ground for 2 min with an ultrasonic probe (Sonics Vibra Cell) (70% amplitude, 130 W, 20 kHz) and then centrifuged at 13,000 rpm for 10 min at room temperature. The collected supernatants underwent final centrifugation at 13,000 rpm for 10 min, and 100 µl of these last supernatants were collected in glass vials and stored at −80°C until analysis.

#### 2.6.3 Identification and quantification of the cyanotoxins

The identification and quantification of the cyanotoxins in the water and fish samples were performed by ultrahigh‐performance liquid chromatography in tandem (Elute UHPLC—Bruker) coupled with high‐resolution mass spectrometry (Compact QTOF—Bruker) (UHPLC–MS/MS). All samples were separated on a C18 stationary phase column (Acclaim RSLC Polar Advance 2, 2.2 µm, Thermo Fisher® 2.1 × 100 mm) along a linear gradient of acidified acetonitrile and ultrapure water acidified to 0.8% and 0.1% formic acid (5:95%, v/v) at 0.3 ml min^−1^ and then analyzed between 50 and 1500 *m/z* in positive BbCID (MS‐MS/MS) mode, alternating at 2 Hz between low‐ and high‐energy MS and MS2 modes, respectively, with a mass accuracy of less than 0.5 ppm. The injection volume and data acquisition time were 4 µl and 21 min, respectively. The elution rate was 0.5 µl min^−1^. The analytical standards of the cyanotoxins investigated were saxitoxin (CAS 35554‐086), anatoxin‐a (CAS 64285‐06‐9), homoanatoxin (CAS 142926‐86‐1), nodularin (CAS 118399‐22‐7), cylindrospermopsin (CAS 143545‐90‐8), and seven variants of microcystins (MCs) (variant MC‐LR, ‐LA, ‐LF, ‐LW, ‐LY, ‐RR and ‐YR corresponding to CAS 101043‐37‐2; CAS 96180‐79‐9; CAS 154037‐70‐4; CAS 157622‐02‐1; CAS 123304‐10‐9; CAS 11755‐37‐4; and CAS 10164‐48‐6, respectively), all certified 1 year, purity >99% (Novakit®). Data were integrated using TASQ 1.1 software (Bruker®). Cyanotoxins were identified according to (i) retention time, (ii) molecular mass, (iii) isotopic pattern, and (iv) diagnostic ions. They were then quantified according to the area under the peak signal determined for analytical standards. *Phormidium favosum* (PMC 240.05), *Raphidiopsis raciborskii* (PMC 99.03), *Aphanizomenon gracile* (PMC 638.10), and *Microcystis aeruginosa* (PMC 728.11) strains were used as positive controls. Except for MC‐RR, MC‐LR, and MC‐YR, other MC structural variants were quantified according to MC‐LR equivalence, calculated from the regression curves of the MC‐LR analytical standard.

### 2.7 Statistical analysis

Coefficients of variation (CVs) were calculated to estimate the extent of variability in the data among the mesocosm triplicates. The CV values obtained for each treatment at each time point were compared by a nonparametric sign test for matched pairs. Three‐way permutational analysis of variance (ANOVA) was performed on the variations in Chl‐a concentrations, richness/diversity (Shannon index) of the phytoplankton community, total cyanobacteria biovolumes, biovolumes of the three dominant cyanobacteria genera, and finally MC concentrations. The three explanatory variables were (i) the station (Débarcadère and Aghien‐Télégraphe), (ii) the five experimental treatments (Control +N, +P, +NP, +NPF), and (iii) the sampling time. We used the “lmp” function from the R package “lmPerm” to fit a linear model with permutations followed by an ANOVA test of the parameters using the “ANOVA” function from the R package “car.”

Principal component analysis (PCA) was used to study the dynamics of the phytoplankton community composition during the experiments. Fisher's exact test was used to compare the proportion of MC+ and MC− fish at the two stations and a Mann–Whitney *U* test was used to compare the MC concentrations in fish intestines at the two stations. Three‐way permutational ANOVA was performed by using R and all other analyses were performed by using XLStat (version 2021‐1‐1‐1095) and PAST (Hammer et al., 2001) software.

## 3 RESULTS

### 3.1 Nutrient concentrations and physicochemical variables

**Figure A1:**
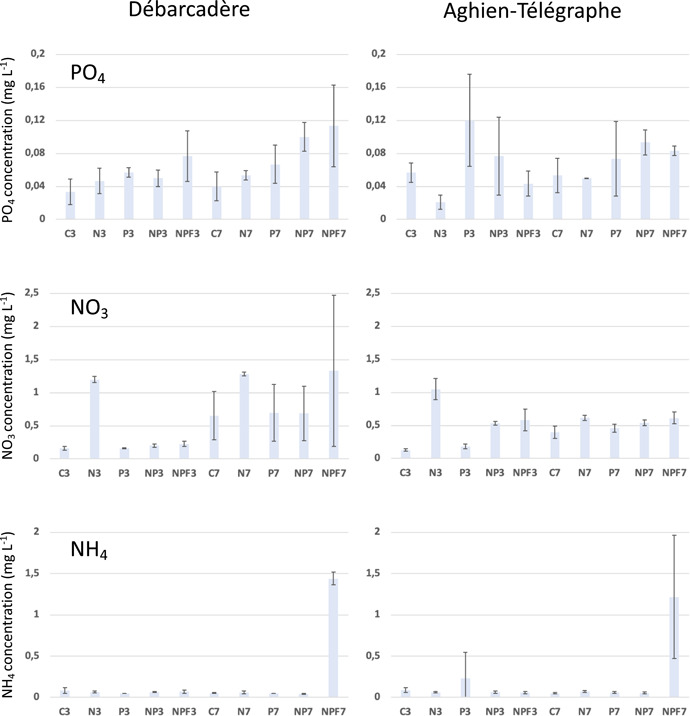
Temporal variations in nutrient concentrations (TN, TP, NO_4_, NH_4_, SRP) on Days 0, 3, and 7 under the five experimental treatments (C, +N +P, +NP, +NPF) at the two stations (Débarcadère and Ahien‐Télégraphe). C, control; D, day; N, +nitrogen; NP, +nitrogen and phosphorus; NPF, +nitrogen, phosphorus, and fish; P, +phosphorus; red arrow, addition of fish to the NPF mesocosms

At D0, the nutrient concentrations (NO_2_, NO_3_, NH_4_, SRP, and TP) were globally similar at the two experimental stations (Débarcadère and Aghien‐Télégraphe) (Table 1). The main difference between the two sites was the higher concentration of TN recorded at Débarcadère than at Aghien‐Télégraphe (1.447 and 1.043 mg L^−1^, respectively). The values of the Chl‐a/DIN (dissolved inorganic nitrogen) and the Chl‐a/TP ratios, which can be used to evaluate whether N, P, or NP are limiting for phytoplankton growth, were similar at the two stations (0.31 and 0.28 for Débarcadère; 1 and 0.8 for Aghien‐Télégraphe, respectively). There was an increase in nitrate concentrations in the N mesocosms at D3 and D7 and a strong increase in ammonium concentrations in the NPF mesocosms at D7 (Figure A1).

**Table 1:** Mean values (n = 3) of nutrient concentrations (mg L^−1^) in the lagoon water on Day 0 at the two experimental stations (Débarcadère and Aghien‐Télégraphe)

	NO_2_ (mg L^−1^)	NO_3_ (mg L^−1^)	NH_4_ (mg L^−1^)	TN (mg L^−1^)	SRP (mg L^−1^)	TP (mg L^−1^)	Chl‐a (mg L^−1^)	Chl‐a/DIN		Chl‐a/TP	
Débarcadère	0.004 ± 0.002^a^	0.120 ± 0.000	0.040 ± 0.026	1.447 ± 0.21	0.006 ± 0.001	0.050 ± 0.000	0.051 ± 0.025	0.31		1	
Aghien‐Télégraphe	0.006 ± 0.002	0.131 ± 0.020	0.042 ± 0.029	1.043 ± 0.046	0.008 ± 0.001	0.063 ± 0.002	0.051 ± 0.003	0.28		0.8	

Abbreviations: Chl‐a, chlorophyll‐a; DIN, dissolved inorganic nitrogen; SRP, soluble reactive phosphorus; TN, total nitrogen; TP, total phosphorus.

^a^ Standard deviation.

**Figure A2:**
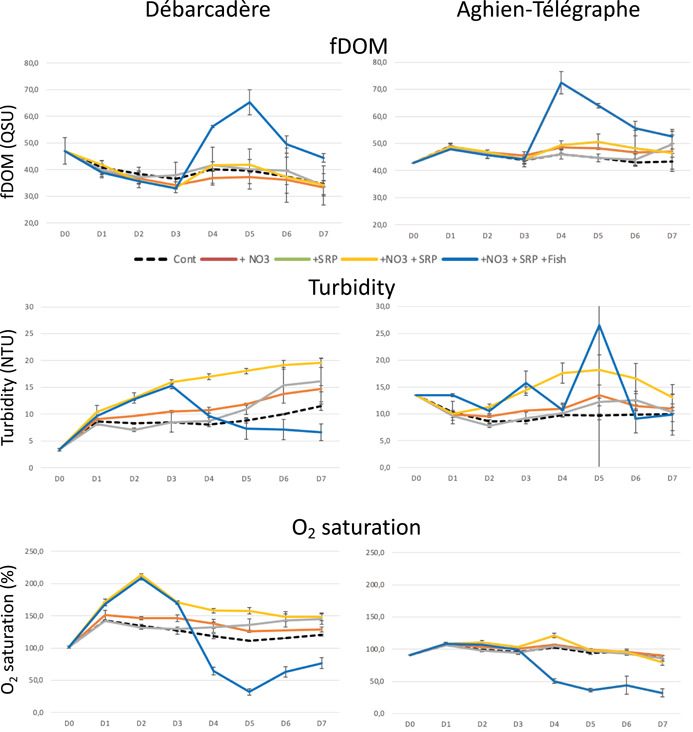
Temporal variations in the fDOM, turbidity, and O_2_ saturation (mean ± *SD*) under the different experimental treatments (C, +N +P, +NP, +NPF) at the Débarcadère (a) and Aghien‐Télégraphe (b) stations. C , control; D, day; N, +nitrogen; NP, +nitrogen and phosphorus; NPF +nitrogen, phosphorus and fish; P, +phosphorus; red arrow, addition of fish to the NPF mesocosms

The water temperature in the mesocosms ranged within the same order at the Aghien‐Télégraphe and Débarcadère stations (30.0 ± 0.3 and 30.7 ± 0.3, respectively). There were no significant variations in the fDOM concentrations during the experiment, except for the NPF mesocosms in which the fDOM concentrations increased after D3 (Figure A2). At theDébarcadère station, the main variations in turbidity were found in the NP and NPF mesocosms, with an increase between D0 and D3, followed by a strong decrease in the NPF mesocosms until D7, while no change occurred from D3 to D7 in the NP mesocosms. The same findings were found at the Aghien‐Télégraphe station, except for the NPF mesocosms displaying more chaotic variations in turbidity. Finally, there was an increase in O_2_ saturation from D0 to D2 in the NP and NPF mesocosms at the Débarcadère station, followed by a dramatic decrease after D3 in the NPF mesocosms (Figure A2). The same decrease in O_2_ saturation was observed in the NPF mesocosms after D3 at the Aghien‐Télégraphe station, while no increase was found in the NP and NPF mesocosms from D0 to D3 (Figure A2).

### 3.2 Dynamics of phytoplankton biomasses (Chl‐a)

**Figure A3:**
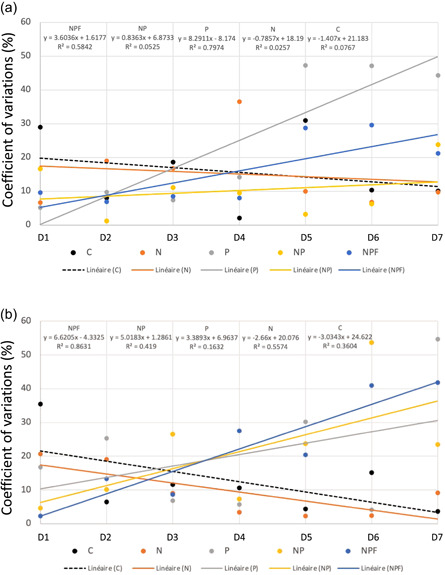
Coefficient of variations of the phytoplankton biomasses (mean Chl‐a ±*SD*) estimated among the triplicate mesocosms from D0 to D7 under the different experimental treatments at the Débarcadère (a) and Aghien‐Télégraphe (b) stations. C, control; N, +nitrogen; NP,  +nitrogen and phosphorus; NPF,  +nitrogen, phosphorus, and fish; P,  +phosphorus

When looking at the global variability in the Chl‐a values among the triplicates (CV values), there was no significant difference (sign test for matched pairs) when comparing data from the two stations. There was an increase in the CV values from D0 to D7 under the P, NP, and NPF treatments but not under the C and N treatments (Figure A3).

**Figure 2:**
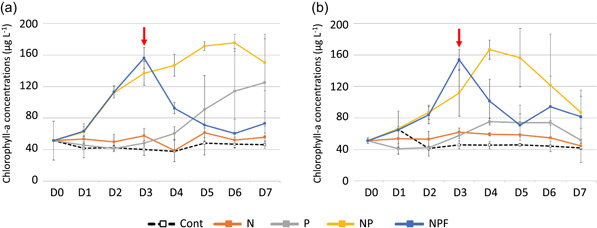
Temporal variations in the phytoplankton biomasses (mean Chl‐a ± *SD*) under the different experimental treatments at the Débarcadère (a) and Aghien‐Télégraphe (b) stations. C, control; D: day; N, +nitrogen; NP, +nitrogen and phosphorus, NPF, +nitrogen, phosphorus and fish; P, +phosphorus; red arrow, addition of fish to the NPF mesocosms

Three‐way permutational ANOVA results (Table A2) showed a significant difference in the Chl‐a concentrations depending on the experimental treatments (*F* = 67.7; *p* < 0.0001), time (*F* = 14.0; *p* < 0.001), and the interaction between treatment and time (*F* = 10.7; *p* < .0001). However, no significant difference was found between the two stations (Table A2). At both stations, there was a strong increase in Chl‐a concentrations until Day 3 in the NP and NPF mesocosms followed by a dramatic decrease in the NPF mesocosms after the addition of fish (Figure 2). The most contrasting difference in the Chl‐a concentrations between these stations was observed for the P treatment with an increase in phytoplankton biomasses from D5 to D7 in Débarcadère but not in Aghien‐Télégraphe, but large CV values were found among the triplicates.

### 3.3 Dynamics of the phytoplankton community

**Figure 3:**
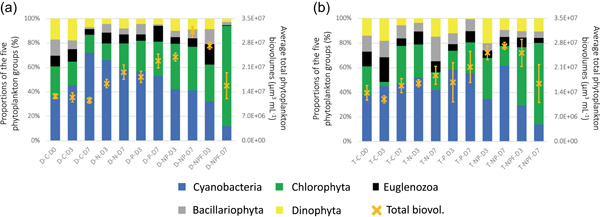
Temporal variations in the mean (±*SD*) total phytoplankton biovolumes (yellow crosses, right Y scale) and the proportions (bars, left Y scale) of the five phytoplankton groups (Cyanobacteria, Chlorophyta, Euglenozoa, Bacillariophyta, and Dinophyta) under the different experimental treatments at the Débarcadère (a) and Aghien‐Télégraphe (b) stations.  C, control; D, day; N, +nitrogen; NP, +nitrogen and phosphorus, NPF, +nitrogen, phosphorus and fish; P, +phosphorus

The temporal variations in the structure of the phytoplankton communities at the phylum level (Cyanobacteria, Chlorophyta, Euglenozoa, Bacillariophyta, and Dinophyta) were globally similar at the two stations (Figure 3). At D0, the phytoplankton communities were mainly composed of Cyanobacteria (35%–40%), Chlorophyta (20%–25%), and other phyla (<40%). At D3, there was an increase in the proportion of Chlorophyta biovolume under the NP and NPF treatments in the Aghien‐Télégraphe experiments and, to a lesser extent, in the Débarcadère experiments, in association with high mean biovolume values. At D7, the most important change was the dominance (>60%) of Chlorophyta and a drastic decrease in Cyanobacteria at the two stations (Débarcadère and Aghien‐Télégraphe) in the NPF mesocosms where fish were added, in association with a decrease in mean biovolume values.

**Figure A4:**
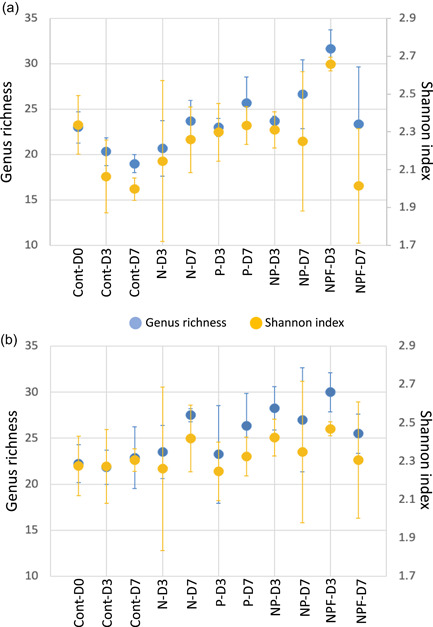
Temporal variations in the richness and diversity (Shannon index) (mean ± *SD*) in the phytoplankton communities under the different experimental treatments at the Débarcadère (a) and Aghien‐Télégraphe (b) stations. C, control; D, day; N, +nitrogen; NP,  +nitrogen and phosphorus; NPF, +nitrogen, phosphorus, and fish; P, +phosphorus

The mean richness values of the phytoplankton communities calculated at the two stations ranged between 19 and 32 genera (Figure A4). Depending on the experimental treatments, there were significant differences in the richness of the phytoplankton communities (*F* = 4.9; *p* < 0.002) (Table A2). Interestingly, the highest richness values were found in the NPF treatments at the two stations at D3, before decreasing at D7.

The mean Shannon index values of the phytoplankton communities were equal to 2.24 (±0.26) and 2.33 (±0.18) in Débarcadère and Aghien‐Télégraphe, respectively (Figure A4). There was a slight but significant difference in the diversity of the phytoplankton communities depending on the time (*F* = 4.81; *p* = 0.03) (Table A2). Similar to the richness, the highest diversity values were found in the NPF treatment at D3, before decreasing at D7.

**Figure 4:**
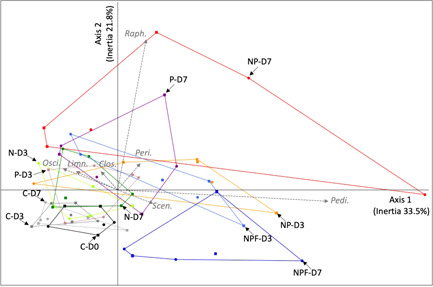
Principal component analysis was performed on the biovolume values recorded for each phytoplankton genus estimated at D0, D3, and D7. Convex hulls were drawn for each mesocosm treatment on each sampling day, taken together at the two stations (Débarcadère, square symbol, and Aghien‐Télégraphe, circle symbol). C, control; *Clos., Closteriopsis/Closterium;* D, day; *Limn., Limnothrix*; N, +nitrogen; NP, +nitrogen and phosphorus, NPF, +nitrogen, phosphorus and fish (NPF); *Osci., Oscillatoria*; P, +phosphorus; *Peri., Peridinium; Pedi., Pediastrum*; *Raph., Raphidiopsis*; *Scen., Scenedesmus*

A principal component analysis (PCA) was then performed on the biovolume values estimated for each phytoplankton genus at D0 (control mesocosms at Aghien‐Télégraphe), D3 (all mesocosms), and D7 (all mesocosms) (Figure 4). The first two eigenvalues of the PCA accounted for >55% of the total variability. The PCA showed the following:

– The structure of the phytoplankton communities in the C mesocosms was very stable during the experiment, as shown by the perfect overlay of the convex hulls of the control mesocosms at D0, D3, and D7.
– Only a few changes occurred in the N mesocosms at D3 and D7 and in the P mesocosms at D3 compared with the C mesocosms, as shown by the partial overlay of their convex hulls with those of the C mesocosms. At D7, in the P mesocosms, more marked changes occurred in the phytoplankton communities of some mesocosms, as shown by the larger surface of their convex hulls (compared with those of the N mesocosms).
– Generally, marked changes occurred in the phytoplankton communities in the NP mesocosms and NPF mesocosms at D3 compared with the C mesocosms.
– There was large heterogeneity in the structure of the phytoplankton communities in the NP mesocosms at D7, as shown by the large surface of the convex hull.
– The phytoplankton communities in the NPF mesocosms at D7 were different from those found in all the other mesocosms; specifically, the surface of the corresponding convex hull was much smaller than that of the NP mesocosms at D7.

**Figure A5:**
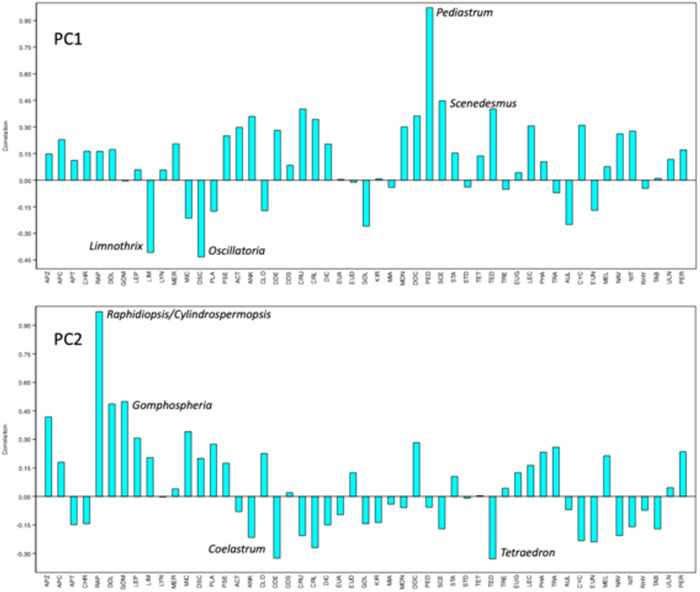
Correlation values between the biovolume values of each phytoplankton genus and the first two components (PC1 and PC2) of the principal component analysis

As shown in Figure A5, the highest correlations recorded between the biovolume values of each genus and the first two components of the PCA were *Pediastrum* (*r* = 0.9), *Scenedesmus* (*r* = 0.15), *Oscillatoria* (*r* = −0.23), and *Limnothrix* (*r* = −0.18) for PC1 and *Raphidiopsis/Cylindrospermopsis* (*r* = 0.94), *Gomphosphaeria* (*r* = 0.50), and *Coelastrum* (*r* = −0.32) for PC2. All these genera belong to the cyanobacteria and Chlorophyta phyla.

### 3.4 Dynamics of cyanobacteria and cyanotoxins

#### 3.4.1 Cyanobacterial dynamics

**Figure A6:**
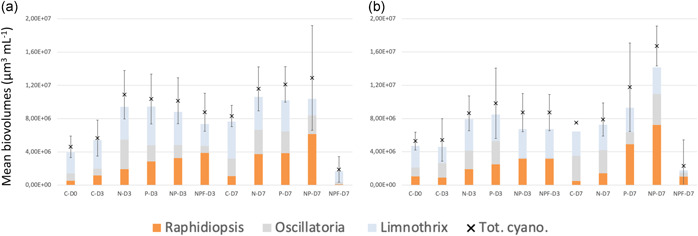
Temporal variations in the mean cyanobacteria biovolumes (±*SD*) and of each of the three dominant cyanobacterial genera under the different experimental treatments at the two stations, Débarcadère (a) and Aghien‐Télégraphe (b). C, control; D, day; N, +nitrogen; NP, +nitrogen and phosphorus; NPF, +nitrogen, phosphorus, and fish (NPF); P, +phosphorus

Treatments, time, and their interaction have a significant impact on the variations in the biovolume values of cyanobacteria (*F* = 9.3, 35.0, and 9.6, respectively; *p* < 0.0001) (Table A2). As shown in Figure A6, there was an increase in the total biovolume values of cyanobacteria during the experiment, except under the NPF treatments at D7. The highest biovolume values were found in the NP mesocosms at D7. In these cyanobacteria communities, three genera (*Limnothrix*, *Oscillatoria*, and *Raphidiopsis*) represented >84% of the total cyanobacterial biovolumes at the two stations (Débarcadère and Aghien‐Télégraphe) (Figure A6).

#### 3.4.2 Cyanotoxins in phytoplankton

Saxitoxin, anatoxin‐a, homoanatoxin, nodularin, and cylindrospermopsin were not detected by UHPLC–MS/MS in any of the analyzed samples. However, MCs were found in all samples. Among them, MC‐RR was the dominant MC variant in all the mesocosms at the two stations. MC‐LR was mostly detected under the NP and NPF treatments from D4 to D7 at Débarcadère station and under nearly all treatments at Aghien‐Télégraphe station (Table A3).

**Figure 5:**
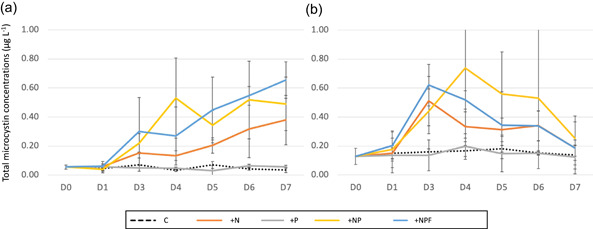
Temporal variations in the total microcystin concentrations (mean ± *SD*) under the different experimental treatments at the Débarcadère (a) and Aghien‐Télégraphe (b) stations. C, control; D, day; N, + nitrogen; NP, +nitrogen and phosphorus; NPF, +nitrogen, phosphorus, and fish (NPF); P, +phosphorus

The average total MC concentrations ranged between 0.07 and 0.53 µg L^−1^ for each mesocosm treatment. As shown in Figure 5, there was an increase in the total MC concentrations in the N, NP, and NPF mesocosms while no variations occurred in the C and P mesocosms. Significant differences in the MC concentrations were found by three‐way permutational ANOVA depending on the treatments (*F* = 28.7, *p* < 0.0001), the time (*F* = 13.4, *p* = 0.0003), and also the station (*F* = 10.0; *p* = 0.001) and their interactions between them (Table A2).

**Figure A7:**
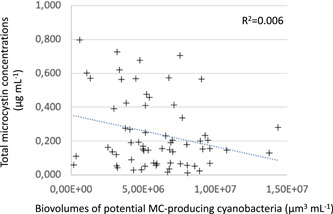
Linear regression between the biovolumes of potential MC‐producing cyanobacteria and the microcystin concentrations at D0, D3, and D7 under the different experimental treatments at the two stations (Débarcadère and Aghien‐Télégraphe)

Finally, there was no significant correlation (*R* ^2^_Pearson_ = 0.006) between the MC concentrations and the sum of the biovolumes of cyanobacterial genera known to be able to potentially produce MCs (*Aphanocapsa, Dolichospermum, Leptolyngbya, Limnothrix, Merismopedia, Microcystis, Oscillatoria, Planktothrix*, and *Pseudanabaena*) (Figure A7).

##### 3.4.2.1 Microcystins in fish

No MC was detected in the livers and muscles of the 34 analyzed fish, while MCs were detected in the intestine of 21 of them (Table 2). These 21 fish containing MC (MC+) were distributed as follows: 4/5 from the control (dissected at D3); 5/15 fish from the NPF mesocosms of Aghien‐Télégraphe (dissected at D7); and 12/14 from the NPF mesocosms of Débarcadère (dissected at D7). This difference between the two stations (Aghien‐Télégraphe and Débarcadère) in terms of the proportion of MC+ fish was statistically significant (Fisher's exact test, *p* = 0.001).

**Table 2:** Microcystin concentrations recorded in the livers, muscles, and intestines of the fish and the status of their intestinal content

	Liver total MCs (µg g^−1^)	Muscle total MCs (µg g^−1^)	Intestine total MCs (µg g^−1^)	Intestine content
Cont 1	0	0	0.26	F
Cont 2	0	0	0.12	F
Cont 3	0	0	0	F
Cont 4	0	0	0.08	F
Cont 5	0	0	0.17	F
Agh‐Tel 1 (NPF3)	0	0	0.06	E
Agh‐Tel 2 (NPF3)	0	0	0.04	E
Agh‐Tel 3 (NPF3)	0	0	0	FC
Agh‐Tel 4 (NPF3)	0	0	0	E
Agh‐Tel 5 (NPF4)	0	0	0	FC
Agh‐Tel 6 (NPF4)	0	0	0	E
Agh‐Tel 7 (NPF4)	0	0	0	FC
Agh‐Tel 8 (NPF4)	0	0	0	FC
Agh‐Tel 9 (NPF4)	0	0	0	FC
Agh‐Tel 10 (NPF8)	0	0	0.06	E
Agh‐Tel 11 (NPF8)	0	0	0.06	E
Agh‐Tel 12 (NPF8)	0	0	0	E
Agh‐Tel 13 (NPF8)	0	0	0	E
Agh‐Tel 14 (NPF8)	0	0	0	E
Agh‐Tel 15 (NPF8)	0	0	0.22	FC
Déb. 1 (NPF3)	0	0	0	FC
Déb. 2 (NPF3)	0	0	0.12	F
Déb. 3 (NPF3)	0	0	0.08	E
Déb.4 (NPF3)	0	0	0.14	FC
Déb. 5 (NPF3)	0	0	0.10	F
Déb. 6 (NPF4)	0	0	0.68	F
Déb. 7 (NPF4)	0	0	0	FC
Déb. 8 (NPF4)	0	0	0.11	FC
Déb. 9 (NPF4)	0	0	0.13	FC
Déb. 10 (NPF8)	0	0	1.00	F
Déb. 11 (NPF8)	0	0	1.43	F
Déb. 12 (NPF8)	0	0	0.51	F
Déb. 13 (NPF8)	0	0	0.41	F
Déb. 14 (NPF8)	0	0	0.42	F

Abbreviations: E, empty; F,  full; FC, few content.

The MC concentrations in the MC + fish ranged from 0.04 µg equiv MC‐LR g^−1^ FW to 1.43 µg equiv MC‐LR g^−1^ FW (mean = 0.30 µg equiv. MC‐LR g^−1^ FW ± 0.36), and these concentrations were higher in the MC+ fish at Débarcadère station than in those at Aghien‐Télégraphe (Mann–Whitney *U* test, *p* < 0.01) (Table 2).

The proportions of fish with a full, empty, or partially filled (few contents) intestine were significantly different between Débarcadère and Aghien‐Télégraphe (Fisher exact test, *p* < 0.001). There were also significant differences in the proportions of MC+ and MC− intestines regarding their content (Fisher's exact test, *p* = 0.014). All the full intestines were MC+, while less than 50% of the empty or partially filled intestines were MC+.

## 4 DISCUSSION

Mesocosm experiments were performed in Lagoon Aghien to assess the potential impact of an increase in nutrient (N and P) concentrations on the phytoplankton community of this aquatic ecosystem. One of the challenges for this 7‐day mesocosm experiment was that the response of the phytoplankton communities to nutrient addition may not be immediate. In previous works performed in microcosms under tropical conditions, very rapid responses (2 days) of the phytoplankton communities were observed after nutrient addition (Leboulanger et al., 2006), as we found in our mesocosms. It is likely that the high water temperature (approximately 30°C) and the high light intensities under tropical latitudes, which allow for a very high growth rate for the phytoplankton, contribute to a large part of the rapid response of these communities in our mesocosms. Under higher latitudes, it could be necessary to perform longer experiments.

The responses of the phytoplanktonic communities to the different experimental treatments were broadly similar in terms of phytoplankton biomass and composition at the two stations. These findings are in agreement with the data obtained during the 17‐month spatial monitoring of some chemical variables and the phytoplankton community in Lagoon Aghien, which did not show any marked differences between the sampling stations during the same period of the dry season (Ahoutou et al., 2021). When considering the repeatability of the Chl‐a data estimated for the triplicate mesocosms under each treatment at each time, the mean CV values were similar (approximately 17%) at the two stations, and these values ranged within the same order as those classically estimated in microcosm/mesocosm experiments (see, e.g., the review by Sanderson, 2002). This result means that the different treatments tested (N, P, NP, and NPF) in this experimental system were the major drivers of the temporal variations recorded in the monitored variables. As expected, we found that there was an overall congruence in the variations occurring in some biological data, such as the Chl‐a concentrations or total phytoplankton biovolumes and those occurring in physicochemical variables, such as the turbidity or O_2_ saturation, particularly at Débarcadère station. The differences in O_2_ saturation values between the two stations were explained by the time of measurement with the probe (approximately 7:00 am in Aghien Télégraphe and 11:00 am in Débarcadère).

Our data showed that there was a significant increase in phytoplankton biomass (estimated by the Chl‐a concentrations and biovolume values of phytoplankton genera) in the mesocosms supplemented with both P and N, which suggests that there is a synergistic effect of P and N addition on phytoplankton growth in Lagoon Aghien. These data are very interesting in relation to the goal of addressing the limitation of primary production in the lagoon because the data on nutrient concentrations did not provide an answer to this question (Ahoutou et al., 2021). Chorus and Spijkerman (2021) suggested that no limitation is caused by nutrients when soluble P concentrations exceed 3–10 µg L^−1^ and dissolved N concentrations exceed 100–130 µg L^−1^. At the beginning of our experiments (Day 0), the soluble P concentrations were approximately 7 µg L^−1^ at both stations, while the total dissolved N concentrations were 160–170 µg L^−1^, which suggested only a potential P limitation. However, as shown in Table 1, the Chl‐a/TP ratio value (~ 1) and the Chl‐a/DIN ratio value (~ 0.3) exceeded the threshold values (P limitation when Chla/TP ratio >0.3; N limitation when Chl‐a/DIN ratio >0.042) defined in Maberly et al. (2020), suggesting a colimitation of phytoplankton growth by P and N. Our data clearly showed that only the addition of both P and N in mesocosms strongly stimulated the growth of phytoplankton communities, which supports the existence of simultaneous P–N colimitation according to the definition proposed by Harpoale et al. (2011). This simultaneous P–N colimitation of primary productivity is very frequent in terrestrial, marine, and freshwater ecosystems, as shown by the meta‐analyses performed by Elser et al. (2007) and Harpole et al. (2011). In the latter paper, it was also shown that simultaneous colimitation by P and N seems to occur more often at higher latitudes and that this kind of limitation tends to be found in ecosystems characterized by low total N and P concentrations. Our data suggest that simultaneous N and P colimitation can also be found in tropical lakes characterized by high levels of these two nutrients.

In addition to this nutrient limitation of the phytoplankton biomass in Lagoon Aghien, our data provide other insights into factors and processes potentially involved in the control of phytoplankton growth in this lagoon. First, the addition of Nile tilapia to the NPF mesocosms at D3 resulted in a drastic reduction in the total phytoplankton biomass, particularly the cyanobacterial biovolumes, at the two stations. This result suggests, in agreement with the works of Torres et al. (2016), that this species is potentially able to control cyanobacteria in tropical lakes. However, it has also been found that depending on the environmental conditions, planktivorous fish may either promote or reduce phytoplankton biomass due to their ability to feed either on phytoplankton or their main grazers (zooplankton) (e.g., Attayde et al., 2010). Second, the high biomasses reached in the mesocosms supplemented with both P and N suggest that there is no light limitation affecting phytoplankton growth in the lagoon, although such a limitation is frequently found in Africa (e.g., Loiselle et al., 2008; Nyakoojo & Byarujali, 2010). Third, we found a decrease in the CV values from D1 to D7 when there was no significant change in the Chl‐a concentrations (C and N treatments) and an increase in these CV values when there was an increase in the Chl‐a concentrations (P, NP, and NPF treatments). This result suggests that other noncontrolled processes/factors may also have an impact on these Chl‐a concentrations when phytoplankton communities are growing in mesocosms.

Variations in phytoplankton biomass were accompanied by changes in the compositions of the phytoplankton communities. The mesocosms supplemented with both P and N were the most heterogeneous at D3 and D7, indicating different trajectories in the evolution of these communities between the replicates. However, the dominant genera found in these NP mesocosms always belonged to Chlorophyta and Cyanobacteria. This dominance of Chlorophyta and Cyanobacteria after the addition of both P and N was also observed in mesocosm experiments performed at Lake Taihu in China (Ma et al., 2015). Knowing that our experiments were performed over a short period (7 days), the most likely explanation for the dominance of Chlorophyta in some NP mesocosms is that they are fast‐growing microorganisms compared with Cyanobacteria (Jensen et al., 1994). Interestingly, the addition of fish to some mesocosms resulted in a dramatic change in the composition of the phytoplankton community, with the disappearance of Cyanobacteria and dominance of Chlorophyta. Moreover, the dispersion of the NPF points at D7 in the PCA was very small compared with that found in the NP mesocosms, suggesting that phytoplankton grazing by fish is a very strong selective pressure compared with all other pressures. In terms of the grazing activity of Nile tilapia, Tesfaye et al. (2020) showed that the diet of adults (length > 10 cm) is mainly macrophytes and phytoplankton, with a preference for Cyanobacteria followed by diatoms and Chlorophyta. Similarly, Semyalo et al. (2011) showed that Cyanobacteria constitute a dominant part of the phytoplankton consumed by Nile tilapia. Consequently, the large dominance of Chlorophyta at D7 in the NPF mesocosms probably resulted from the consumption of Cyanobacteria and/or from the more rapid growth of Chlorophyta.

In addition to the impact of fish on the phytoplankton biomass, our data showed that fish had a direct impact on the fDOM concentrations, as it has already been observed in aquaculture systems in which water exchange is low (Yamin et al., 2017). In such systems, humic substances result from the degradation of organic matter contained in fish feces. Similarly, we found that there was a strong increase in ammonia concentrations at D7 in the NPF mesocosms and that ammonia production/excretion by fish is a well‐known process (see e.g., the review by Ip & Chew, 2010). Finally, fish respiration may also explain a large part of the low O_2_ saturation values (approximately 50%) from D4 to D7 in the NPF mesocosms.

MCs were detected in numerous mesocosms but no other cyanotoxins (cylindrospermopsins, saxitoxins, anatoxin‐a, homoanatoxin, and nodularins) were observed despite the presence of cyanobacterial genera potentially able to produce several of these toxins. These data are in agreement with those reported in the review papers by Mowe et al. (2015) and Svircev et al. (2019), showing that MCs are the most frequent cyanotoxins found in tropical Africa. They also confirmed that *Raphidiopsis*, which was one of the dominant genera in some mesocosms, very infrequently produces cylindrospermopsins and saxitoxins in Africa.

No significant correlation was found between the variations occurring in the biomasses of potential MC‐producing cyanobacteria and those occurring in MC concentrations. This lack of relationship was already described by Mowe et al. (2015) in tropical African blooms as opposed to tropical Asian or American blooms. However, our findings showed that a similar increase was found in the MC concentrations in the mesocosms supplemented with N, alone or associated with P, while very small quantities were found in the C and P mesocosms during the entire course of the experiment. Knowing that there was no significant increase in the potential MC‐producing cyanobacterial biomass in the mesocosms supplemented with N, nitrogen may stimulate MC production by MC‐producing Cyanobacteria. This finding is in agreement with several studies (e.g., Chaffin et al., 2018; Horst et al., 2014; Wagner et al., 2021), which showed that increasing the N supply increased MC cell quotas and concentrations in both *Microcystis* and *Planktothrix* cultures and natural cyanobacterial blooms.

Surprisingly, high MC concentrations were recorded at D7 in the NPF mesocosms at the Débarcadère station, while there was a strong decrease in the Chl‐a concentrations and cyanobacterial biovolumes. In contrast, very low MC concentrations were recorded in the NPF mesocosms at the Aghien‐Télégraphe station. This result might be explained by the fact that at Débarcadère station, most of the fish had full intestines, containing small amounts of MCs, while at Aghien‐Télégraphe station, most of the fish had empty or partially filled intestines with few MCs. Given that Lu et al. (2006) showed that the digestion efficiency of Nile tilapia in terms of cyanobacteria ranged from 58% to 78%, it can be assumed that this partial digestion results in the production of feces containing partially destroyed cyanobacterial cells with their MCs. We have no clear hypothesis explaining the difference in the fish intestine contents found at the two stations. However, it should be noted that due to the windy conditions observed during the experiment at Aghien‐Télégraphe station, mesocosms at this station were much rougher than those at Débarcadère station, which may have influenced fish behavior and consequently their intestine content. Finally, some bacteria, fungi, zooplankton, and plants can biodegrade MC in water and fish intestines (see e.g., the review by Li et al., 2017), but we do not have any data to discuss the impact of this potential process in our results.

All these findings are interesting to discuss regarding the issue of controlling cyanobacterial blooms in the lagoon and limiting human exposure to cyanotoxins. Numerous studies have been published in the past 20 years on the management of nutrients for the control of cyanobacterial blooms with the important question of choosing to target only P or both P and N (e.g., Chorus & Spijkerman, 2021; Paerl et al., 2020). Our findings showed that (i) in some P mesocosms, N_2_‐fixing cyanobacteria (in particular *Raphidiopsis*) were able to grow and (ii) N addition seemed to stimulate MC production, suggesting that the control of both N and P is needed in Lagoon Aghien. However, in the local context of this lagoon, this reduction in the N and P loads will be difficult to achieve because, as shown by Koffi et al. (2019), nutrient inputs are likely to increase in the near future due to the expansion of urban areas within the watershed of the lagoon and the lack of sewage systems and treatment.

Finally, while ex situ and in situ mesocosm experiments are frequently used in northern countries with the goal of better understanding the responses of aquatic ecosystems to various stresses, few studies based on these approaches have been performed in freshwater Sub‐Saharan ecosystems (i.e., Buxton et al., 2020; Masese et al., 2020; Melack et al., 1982; Troussellier et al., 2005). The main reasons for this are probably that the equipment is considered quite expensive, and its implementation may cause difficulties with local populations (e.g., theft or degradation of equipment). This study shows that it is possible to develop mesocosm systems at a moderate cost that are also easy to transport and construct in the field. As already observed during a pilot study on citizen monitoring of Lagoon Aghien (Mitroi et al., 2020), we also showed that it is possible to benefit from active local population support during experiments, in particular, support for the installation of the mesocosms and their monitoring when scientific teams were not present on site. These actions occur when these experiments are prepared by these populations.

## AUTHOR CONTRIBUTIONS

**Mathias K. Ahoutou**: Investigation (supporting); methodology (supporting); formal analysis (supporting). **Eric K. Yao**: Investigation (supporting); methodology (supporting); formal analysis (supporting). **Rosine Y. Djeha**: Investigation (supporting). **Mamadou Kone**: Methodology (supporting); formal analysis (supporting). **Kevin Tambosco**: Methodology (supporting). **Charlotte Duval**: Investigation (supporting); methodology (supporting); formal analysis (supporting). **Sahima Hamlaoui**: Investigation (supporting); methodology (supporting); formal analysis (supporting). **Cécile Bernard**: Conceptualization (equal); investigation (supporting); methodology (supporting); formal analysis (supporting); writing—review & editing (equal). **Marc Bouvy**: Conceptualization (equal); investigation (supporting); formal analysis (supporting); writing—review & editing (equal). **Benjamin Marie**: Methodology (supporting); formal analysis (supporting). **Bernard Montuelle**: Conceptualization (equal); investigation (supporting); formal analysis (supporting). **Marc Troussellier**: Conceptualization (equal); investigation (supporting); methodology (supporting); formal analysis (supporting); writing—review & editing (equal). **Felix K. Konan**: Conceptualization (equal). **Julien K. Coulibaly**: Conceptualization (equal); project administration (supporting). **Mireille Dosso**: Project administration (supporting). **Jean‐François Humbert**: Investigation (supporting); methodology (supporting); Formal analysis (supporting); conceptualization (equal); funding acquisition (lead); writing—original draft (equal); writing—review & editing (equal); project administration (lead). **Catherine Quiblier**: Investigation (supporting); methodology (supporting); formal analysis (supporting); conceptualization (equal); writing—original draft (equal); writing—review & editing (equal).

## CONFLICTS OF INTEREST

None declared.

## ETHICS STATEMENT

None required.

## Data Availability

All data analyzed during this study are included in the article except for the supplemental table available in Zenodo at https://doi.org/10.5281/zenodo.6351666 (Biovolumes data of the phytoplankton communities in mesocosm experiments).

## APPENDIX A

See Figures A1, A2, A3, A4, A5, A6, A7 and Tables A1, A3.

**Table A1:** Main characteristics of the two stations

	Débarcadère	Aghien‐Télégraphe
Water temperature (°C)	30.1	30
Turbidity (NTU)	8.4	13.5
O_2_ concentrations (mg L^−1^)	7.7	8.1
fDOM (QSU)	46.5	42.8

Abbreviation: fDOM, fluorescent dissolved organic matter

**Table A2:** Summary data from ANOVA performed on chlorophyll‐a concentrations, richness, and diversity (Shannon index) of the phytoplankton communities, cyanobacteria biovolumes, and microcystin concentrations

Variations in chlorophyll‐a concentrations during the experiments				
	*DF*	Sum of squares	*F* value	*P* >* F*
Treatment	4	175,158	67.7	**<2.2e**–**16**
Time	1	9055	14.0	**0.0002**
Station	1	1752	2.7	0.10
Treatment x time	4	27,550	10.7	**<8.7e**–**08**
Treatment x station	4	6456	2.5	**0.04**
Time x station	1	3293	5.1	**0.02**
Treatment x time x station	4	7114	2.7	**0.03**
Residuals	190	122,857		

Variations in the richness and diversity (Shannon) of the phytoplankton communities during the experiments				
Richness				
	*DF*	Sum of squares	*F* value	*P* > *F*
Treatment	4	199.1	4.9	**0.002**
Time	1	19.9	1.9	0.17
Station	1	26.7	2.6	0.11
Treatment x time	4	20.9	0.5	0.7
Treatment x station	4	19.8	0.5	0.7
Time x Station	1	4.3	0.4	0.5
Treatment x Time x station	4	38.0	0.9	0.4
Residuals	70	713.9		

Diversity (Shannon)				
	*DF*	Sum of squares	*F* value	*P* > *F*
Treatment	4	0.23	1.38	0.25
Time	1	0.20	4.81	**0.03**
Station	1	0.14	3.50	0.06
Treatment x time	4	0.09	0.56	0.69
Treatment x station	4	0.07	0.47	0.75
Time x station	1	0.04	1.05	0.31
Treatment x time x station	4	0.08	0.50	0.73
Residuals	70	2.92		

Variations in cyanobacteria biovolume values during the experiments				
	*DF*	Sum of squares	*F* value	*P* > *F*
Treatment	4	2.8e + 14	9.3	**4.5e**–**06**
Time	1	2.6e + 14	35.0	**1.1e**–**07**
Station	1	895911254424	0.1	0.7
Treatment x time	4	2.9e + 14	9.6	**2.9e**–**06**
Treatment x station	4	17852073956242	0.6	0.7
Time x Station	1	1642443167915	0.2	0.6
Treatment x time x station	4	23682637979715	0.8	0.5
Residuals	70	5.3e + 14		

Variations in microcystin concentrations during the experiments				
Source	*DF*	Sum of square	*F* value	*P* > *F*
Treatment	4	2.98	28.7	**<2.2e**–**16**
Time	1	0.35	13.4	**0.0003**
Station	1	0.26	10.0	**0.001**
Treatment x time	4	0.29	2.8	**0.02**
Treatment x station	4	0.09	0.89	0.47
Station x Time	1	0.35	13.3	**0.0003**
Treatment x station x time	4	0.31	2.9	**0.02**
Residuals	159	4.13		

*Note*: Significant *P* values (*P* < 0.05) in bold.

Abbreviation: ANOVA, analysis of variance.

**Table A3:** Microcystin concentrations during the experiment in the 15 mesocosms at the two stations (Débarcadère and Aghien‐Télégraphe)

Débarcadère Aghien‐Télégraphe							
		MC‐LR	MC‐RR	Total MC	MC‐LR	MC‐RR	Total MC
Day	Mesocosm	µg L^−1^	µg L^−1^	µg L^−1^	µg L^−1^	µg L^−1^	µg L^−1^
DO	C1	0	0.043	0.043	nd	nd	nd
	C2	0	0.055	0.055	0.050	0.119	0.169
	C3	0	0.070	0.070	0.026	0.063	0.089
D1	C1	0	0.052	0.052	0.044	0.111	0.155
	C2	0	0.013	0.013	0.081	0.102	0.184
	C3	0	0.073	0.073	0.027	0.084	0.111
	N1	0	0.060	0.060	0.120	0.085	0.205
	N2	0	0.049	0.049	0.013	0.086	0.099
	N3	0	0.066	0.066	0.022	0.124	0.146
	P1	0	0.043	0.043	nd	nd	nd
	P2	0	0.098	0.098	0.094	0.125	0.219
	P3	0	0.032	0.032	0.023	0.028	0.051
	NP1	0	0.044	0.044	0.109	0.139	0.248
	NP2	0	0.045	0.045	0.090	0.164	0.254
	NP3	0	0.034	0.034	0	0.030	0.030
	NPF1	0	0.089	0.089	0.104	0.161	0.266
	NPF2	0	0.026	0.026	0.021	0.076	0.097
	NPF3	0	0.070	0.070	0.119	0.129	0.248
D3	C1	0	0.029	0.029	0.021	0.142	0.162
	C2	0	0.120	0.120	0.056	0.080	0.137
	C3	0	0.066	0.066	0.052	0.127	0.178
	N1	0	0.157	0.157	0.145	0.267	0.411
	N2	0	0.156	0.156	0.255	0.452	0.706
	N3	0	0.146	0.146	0.179	0.234	0.413
	P1	0	0.024	0.024	0.068	0.124	0.193
	P2	0	0.065	0.065	0.039	0.162	0.201
	P3	0	0.057	0.057	0	0.013	0.013
	NP1	0	0.276	0.276	0.167	0.309	0.477
	NP2	0	0.234	0.234	0.198	0.375	0.573
	NP3	0	0.149	0.149	0	0.269	0.269
	NPF1	0.319	0.250	0.568	0.394	0.333	0.727
	NPF2	0	0.144	0.144	0.214	0.464	0.677
	NPF3	0	0.191	0.191	0.137	0.320	0.457
D4	C1	0	0.034	0.034	0.057	0.107	0.164
	C2	0	0.023	0.023	0.075	0.130	0.205
	C3	0	0.039	0.039	0.013	0.116	0.129
	N1	0	0.121	0.121	0.092	0.304	0.396
	N2	0	0.171	0.171	0.060	0.154	0.214
	N3	0	0.110	0.110	0.139	0.252	0.391
	P1	0	0.055	0.055	0.122	0.166	0.287
	P2	0	0.049	0.049	0.067	0.128	0.195
	P3	0	0.038	0.038	0	0.110	0.110
	NP1	0.414	0.331	0.745	0.140	0.296	0.436
	NP2	0	0.220	0.220	0.074	0.407	0.480
	NP3	0.387	0.240	0.627	0.646	0.650	1.296
	NPF1	0	0.156	0.156	0.340	0.247	0.588
	NPF2	0.403	0.098	0.501	0.223	0.241	0.464
	NPF3	0	0.152	0.152	0.260	0.242	0.501
D5	C1	0	0.072	0.072	0.035	0.123	0.158
	C2	0	0.045	0.045	0.076	0.129	0.205
	C3	0	0.093	0.093	0.072	0.109	0.181
	N1	0	0.152	0.152	0.176	0.222	0.398
	N2	0	0.205	0.205	0.077	0.159	0.237
	N3	0	0.256	0.256	0.056	0.247	0.302
	P1	0	0.0004	0.000	0.099	0.092	0.191
	P2	0	0.020	0.020	0.079	0.170	0.249
	P3	0	0.070	0.070	0	0.006	0.006
	NP1	0	0.319	0.319	0.056	0.259	0.315
	NP2	0.292	0.163	0.455	0.380	0.502	0.881
	NP3	0	0.255	0.255	0.145	0.334	0.479
	NPF1	0.415	0.192	0.607	0.030	0.058	0.089
	NPF2	0	0.191	0.191	0.320	0.214	0.534
	NPF3	0.356	0.194	0.550	0.217	0.193	0.410
D6	C1	0	0.034	0.034	0.061	0.096	0.157
	C2	0	0.049	0.049	0.056	0.108	0.164
	C3	0	0.042	0.042	0.052	0.083	0.135
	N1	0.290	0.254	0.544	0.122	0.208	0.331
	N2	0	0.200	0.200	0.025	0.219	0.244
	N3	0	0.209	0.209	0.204	0.242	0.447
	P1	0	0.054	0.054	0.014	0.129	0.143
	P2	0	0.075	0.075	0.063	0.099	0.161
	P3	0	0.064	0.064	0	0.147	0.147
	NP1	0.435	0.315	0.749	0.159	0.230	0.389
	NP2	0.351	0.226	0.577	0.404	0.665	1.069
	NP3	0	0.226	0.226	0.048	0.081	0.130
	NPF1	0.396	0.189	0.584	0.162	0.061	0.222
	NPF2	0.341	0.130	0.471	0.233	0.186	0.419
	NPF3	0.361	0.223	0.584	0.244	0.130	0.374
D7	C1	0	0.017	0.017	0.067	0.138	0.205
	C2	0	0.056	0.056	0.048	0.088	0.136
	C3	0	0.035	0.035	0.025	0.047	0.072
	N1	0.302	0.264	0.566	0.037	0.118	0.155
	N2	0	0.338	0.338	0	0.152	0.152
	N3	0	0.232	0.232	0.035	0.215	0.250
	P1	0	0.051	0.051	0.073	0.119	0.191
	P2	0	0.052	0.052	0.052	0.095	0.147
	P3	0	0.069	0.069	0	0.031	0.031
	NP1	0.355	0.267	0.621	0.051	0.155	0.206
	NP2	0.309	0.262	0.571	0.148	0.276	0.424
	NP3	0	0.280	0.280	0.019	0.110	0.129
	NPF1	0.444	0.353	0.797	0.074	0.036	0.110
	NPF2	0.439	0.164	0.603	0.267	0.124	0.391
	NPF3	0.386	0.178	0.564	0.011	0.049	0.060

Abbreviations: C, control; D, day; N, +nitrogen, nd, no data; NP, +nitrogen and phosphorus; NPF, +nitrogen, phosphorus, and fish (NPF); P, +phosphorus.
